# Compound Motor Action Potentials During a Modest Nerve Crush

**DOI:** 10.3389/fncel.2022.798203

**Published:** 2022-03-30

**Authors:** Mohammed Nazmy Hamad, Nickolas Boroda, Diego Barragan Echenique, Raymond A. Dieter, Farid M. L. Amirouche, Mark H. Gonzalez, James M. Kerns

**Affiliations:** ^1^Department of Orthopedic Surgery, University of Illinois Chicago, Chicago, IL, United States; ^2^Hines Veterans Affairs Hospital Research Service, Hines, IL, United States

**Keywords:** nerve crush with feedback, instrumented forceps with force transducer, axonotmesis, controlled crush parameters, controlled compression parameters, force-impulse, rat sciatic tibial nerve crush injury, compound motor action potential (CMAP)

## Abstract

Nerve crush injury results in axonotmesis, characterized by disruption of axons and their myelin sheaths with relative sparing of the nerve’s connective tissue. Despite the widespread use of crush injury models, no standardized method for producing these lesions has been established. We characterize a crush model in which a narrow forceps is used to induce a modest and controlled compressive injury. The instantaneous compound motor action potential (CMAP) is monitored *in situ* and in real-time, allowing the characterization of neuromuscular response during and after injury. The tibial nerves of 11 anesthetized rats were surgically isolated. After the placement of electrodes, CMAPs were elicited and registered using a modular-data-acquisition system. Dumont-#5 micro-forceps were instrumented with a force transducer allowing force measurement *via* a digital sensor. Baseline CMAPs were recorded prior to crush and continued for the duration of the experiment. Nerve crushing commenced by gradually increasing the force applied to the forceps. At a target decrease in CMAP amplitude of 70%–90%, crushing was halted. CMAPs were continually recorded for 5–20 min after the termination of the crushing event. Nerves were then fixed for histological assessment. The following post-crush mean values from 19 trials were reported: peak CMAP amplitude decreased by 81.6% from baseline, duration of crush was 17 sec, rate of applied force was 0.03 N/sec, and maximal applied force was 0.5 N. A variety of agonal phenomena were evident post-lesion. Following the initial decrease in CMAP, 8 of 19 trials demonstrated a partial and transient recovery, followed by a further decline. Thirteen trials exhibited a CMAP amplitude near zero at the end of the recording. Twelve trials demonstrated a superimposed EMG background response during and after the crush event, with disappearance occurring within 4–8 min. Qualitative histology assessment at the lesion site demonstrated a correspondence between CMAP response and partial sparing of nerve fibers. By using a targeted decline in CMAP amplitude as the endpoint, researchers may be able to produce controlled, brief, and reproducible crush injuries. This model can also be used to test interventions aimed at enhancing subsequent regeneration and behavioral recovery.

## Introduction

Axonotmesis is a peripheral nerve lesion paradigm characterized by disruption of axons and their myelin sheath with relative sparing of the nerve’s supporting connective tissues. Preservation of these supporting structures allows regenerating axonal growth cones to re-grow through their original paths and to re-innervate their distal targets after injury (Seddon, 1943; Sunderland, 1951; Campbell, 2008). This confers an excellent prognosis. Hence, following axonotmesis, nerves typically achieve complete or nearly complete restoration of motor and sensory function by 4–5 postoperative weeks (De Koning et al., 1986; Malushte et al., 2004). This feature of axonotmesis has made it invaluable in the study of nerve degeneration and regeneration (Chung et al., 2014; Dun and Parkinson, 2018).

Experimentally, axonotmesis is usually produced by maximally applying mechanical pressure to the nerve *via* micro-forceps (Kurtoglu et al., 2005; Fan et al., 2015; Ni et al., 2017; Suzuki et al., 2017), clamps (Zhang et al., 2013; Yuce et al., 2015; Korkmaz et al., 2016), tourniquets (Chen et al., 1992), or other instruments (Sarikcioglu et al., 2007; Feng and Yuan, 2015; Hei et al., 2016). Despite published attempts to produce more consistent lesions (Beer et al., 2001), no standardized method for inducing axonotmesis has been established (Varejao et al., 2004; Ronchi et al., 2009). Studies vary widely with regards to the instrument used, the duration of force applied, the lesion size, and the magnitude/reproducibility of the resulting lesion (Tos et al., 2009; Alvites et al., 2018). Consequently, the lack of a defined standard often makes comparisons between different experimental investigations difficult.

The parameters which characterize the severity of a crush lesion are described by the force-impulse (F-i), which is the product of the force and duration of the compressive pressure applied to the nerve (Liu et al., 2020). The extent of nerve regeneration has been shown to be dependent on the F-i of the trauma sustained to the nerve (Chen et al., 1992; Sarikcioglu et al., 2007). Compound motor action potentials (CMAPs) have been used to quantify neuromuscular function following induced nerve injury (Robinson, 2000; Sta et al., 2014; Vannucci et al., 2019). This electrophysiological measure reflects summations of the evoked action potentials generated by motor units as measured by electrodes inserted at the target muscles (Menorca et al., 2013; Bhatt et al., 2016).

In the present study, we investigate whether CMAP can be used as a reliable endpoint for conducting nerve crush injuries and whether this may offer a more standardized approach for cross-comparison of nerve injuries. To characterize all the parameters of the induced crush injury, we used instrumented micro-forceps to allow real-time monitoring and recording of the F-i applied while conducting the injury. The applied F-i is controlled by the investigator based on feedback from the instantaneous CMAP, which is used as a surrogate for functional decline of motoneuron-activity (Navarro and Udina, 2009; Navarro, 2016). We hypothesize that if nerves are crushed by a graded force to a targeted decline in CMAP amplitude, then the resulting lesions will exhibit similar degrees of injury, and the injury will be highly reproducible. Using our proposed technique, we characterize the electrophysiological changes of neuromuscular response during and immediately following crush injury. Qualitative histology is also used to characterize and provide structural confirmation of the degree of nerve injury.

## Materials and Methods

### Experimental Animals

Sprague Dawley female rats (*n* = 11) were used in this study. Two rats were housed per cage at the University of Illinois Biological Research Laboratory Vivarium under an *ad libitum* diet with a 12-h light/dark cycle. The rats were acquired at a weight between 175 and 200 g (Charles River Laboratory; Chicago, IL, United States) and allowed to acclimate until they reached a weight of approximately 250–275 g. Animals were numbered and weighed before undergoing surgery. All experimental procedures were in accordance with the National Institute of Health Guide for the Care and Use of Laboratory Animals and the University of Illinois at Chicago Institutional Care and Use Committee.

### Anesthesia and Surgical Procedure

Prior to the procedure, the animals were anesthetized with an intraperitoneal injection (ketamine HCL 90 mg/kg and xylazine 10 mg/kg) and given a subcutaneous injection of buprenorphine SR Lab (1.0 mg/kg) for acute pain management. After shaving the operative area of the experimental limbs, rats were placed on a heating pad and a rectal probe was inserted to monitor body temperature for the duration of the procedure. A solution of 10% povidone iodine was applied in triplicate to the surgical area, followed by a 70% alcohol solution to sterilize the field. A sterile drape was applied over the area and a chevron incision was created directly caudal to the femur and the tibia of the rat. After retracting the skin overlying the incision, the exposed biceps femoris muscle was split using a lateral approach, exposing the sciatic nerve as well as both heads of the gastrocnemius muscle. Using micro-instruments, the sciatic nerve was isolated from the surrounding tissue. The tibial, peroneal, and sural fascicles were isolated. To avoid recording CMAPs from the peroneal and sural nerve fascicles, these branches were transected. The nerve crush was performed while monitoring and recording the CMAP. Following the procedure, the incision was closed with a running stitch using a 4.0 Nylon suture (Ethicon, Inc.; Raritan, NJ, United States). The procedure was repeated on the animal’s contralateral side, allowing a total of 22 crush lesion trials. The animals were euthanized while fully anesthetized and unconscious following our institution’s guidelines.

### CMAP Recordings

**Figure 1:**
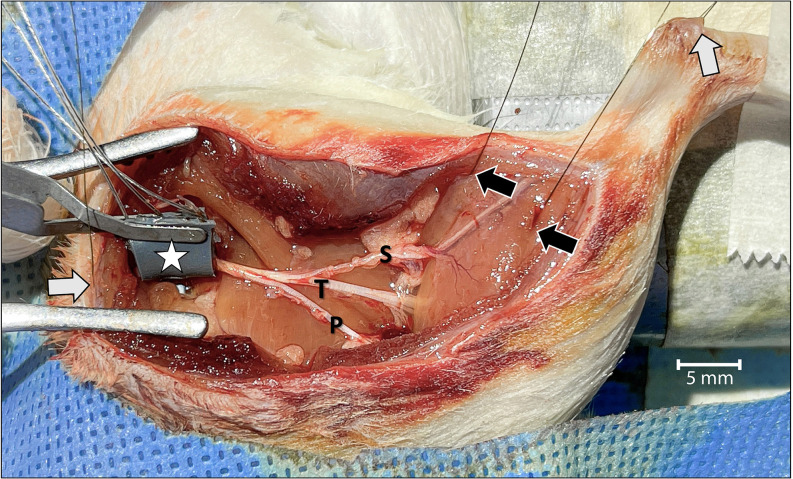
Surgical view of the left leg showing the acute dissection and the methods used to establish two-channel CMAP recording and nerve stimulation. Wire-hook recording electrodes were inserted into the belly of the lateral and medial gastrocnemius muscles (black arrows) and Achilles tendon (white arrow in the upper right). A grounding electrode was placed under the skin of the back of the rat (white arrow on the left). The bipolar stimulating nerve cuff electrode with its connecting wires was wrapped around the proximal sciatic nerve at the level of the sciatic notch (star). To isolate motor unit activation corresponding only to the tibial (T) nerve, and to prevent signal interference, the sural (S) and peroneal (P) nerves were cut. The tibial nerve was crushed just distal to the point of sural nerve take-off. For orientation, the rostral direction is down to the left.

Continuous two-channel CMAP recordings were obtained by placing wire-hook bipolar electrodes (EMG Hook Electrodes, Model EMT-2-30: Microprobes, Inc.; Gaithersburg, MD, United States) at four different locations (Figure 1). One recording electrode each was placed at the muscle belly of the lateral and medial heads of the gastrocnemius, and two corresponding electrodes were placed in the Achilles tendon. A ground electrode was placed under the skin of the back of the rat. A bipolar stimulating cuff electrode was placed around the sciatic nerve proximal to the level of the trifurcation in order to complete the circuit.

**Figure 2:**
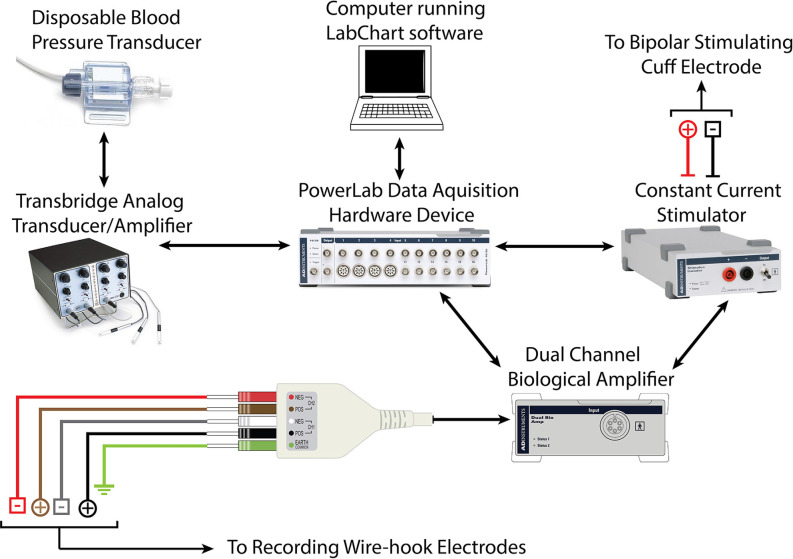
Schematic demonstrating hardware requirements for recording CMAP and force-impulse of crush lesion using forceps instrumented with a force transducer.

Recordings were obtained using a Power Lab Modular Data Acquisition System, LabChart recording software, and dual-channel EMG Bioamplifier (ADInstruments, Inc.; Dunedin, New Zealand). CMAP data were processed through a low pass filter at 1 KHz, and a high pass filter at 1 Hz. An active main filter was applied with a 3 sec delay to eliminate 60 Hz interference once recordings start (Figure 2).

**Figure 3:**
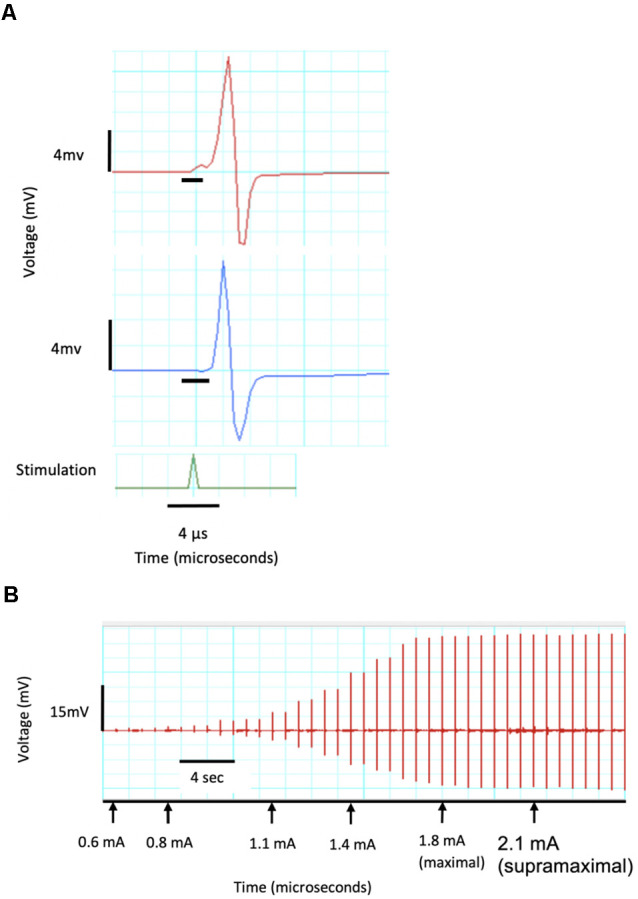
CMAP recordings demonstrating a current response test from a single channel. **(A)** Recording of two CMAPs with peaks of 8.5 and 11 mV as well as a delay of 0.03 and 0.02 μs which are identified by the black horizontal crossbars. **(B)** Current response curve showing threshold CMAP at a stimulating current of 0.6 mA, maximal response at 1.8 mA and 120% maximal (supramaximal) response at 2.1 mA and used in all further CMAP tests.

Each motor unit has a different threshold stimulus intensity at which it can be activated. To ensure that all motor units of the target muscles were activated and accounted for in the CMAP data obtained, a current-response curve was generated prior to each experimental trial (Maathuis et al., 2011). The sciatic nerve was stimulated with electrical pulses of gradually increasing current intensities ranging from subthreshold to supramaximal. This was implemented using a constant-current stimulator (ADInstruments, Inc.; Dunedin, New Zealand) which induced stimulation pulses of 0.05 ms durations at increments of 0.1 mA. The current which corresponded to 150% of the maximal CMAP millivolt peak was used for nerve stimulation for the respective experiment (Figure 3).

CMAP recordings were initiated prior to inducing the nerve lesion in order to capture the baseline CMAP amplitudes before the trauma. Recordings were implemented continuously for the duration of the experiment and for 5–20 min after the termination of the crushing event. This allowed the CMAP response to be monitored in real-time *in situ*.

### Nerve Crush Using Instrumented Micro-forceps

**Figure 4:**
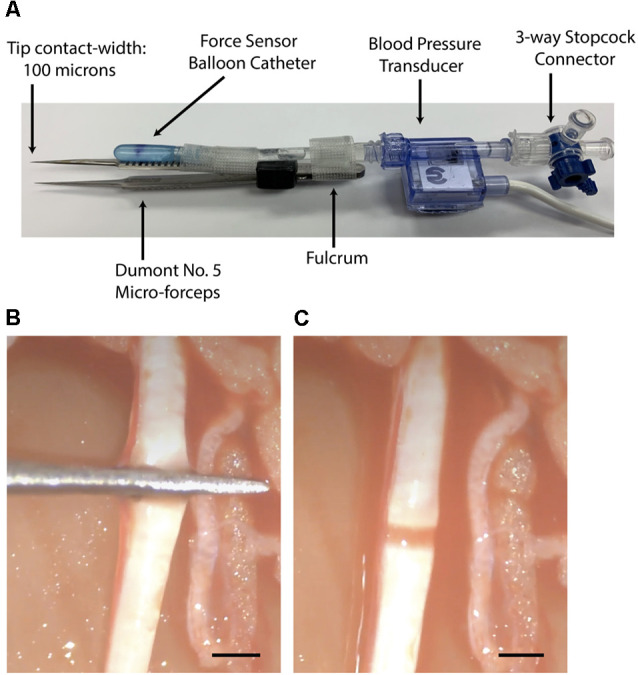
**(A)** Dumont No. 5 micro-forceps and interface shown. **(B)** View of the tibial nerve during crushing with the instrumented micro-forceps. **(C)** View of the nerve crush lesion ~5 sec after crush giving a narrow and translucent appearance. Bars = 1 mm.

A precise and controlled compressive nerve lesion was delivered to the tibial nerve using a Dumont No. 5 micro-forceps (INOX 0508-L5-PO, Catalog No. 10-001-130, Hatfield, PA, United States: Electron Microscopy Sciences; Figure 4A). The absolute tip of the forceps had a contact-width of 0.1 mm, however, we conducted lesions at a point ~2 mm from the tip. This point had a contact width of ~0.3 mm, as measured using a digital caliper. Forceps were instrumented with a force transducer to allow real-time measurement of the applied compressive pressure. The force transducer consisted of a thin-walled water-filled balloon made of relatively inelastic low-density polyethylene attached to a disposable blood pressure transducer (Blood Pressure Transducer and Cable, Model BLPR2, Sarasota, FL, United States: World Precision Instruments Inc.). The balloon was adopted from a modified commercially-available rectal catheter (Urodynamic Rectal Catheter, Model 023121, Orangeburg, NY, United States: Laborie Inc.) in which the distal end was filled with a silastic tube, sealed with silastic, and secured with 0-grade suture thread. The sealed balloon, its associated tubing, and the pressure transducer were filled with water *via* an attached three-way connector in order to improve the fidelity of the recordings. The assembled device was connected to a bridge-circuit transducer, quad-channel amplifier (4-Channel Transducer Amplifier, Model TBM4M, Sarasota, FL, United States: World Precision Instruments Inc.), and to the PowerLab Modular Data Acquisition System to allow monitoring and recording of applied force (Figure 2). The limited elasticity of the balloon resulted in an accurate recording of finger pressure applied and force transmitted to the micro-forceps.

The balloon of the force transducer was fixed to one side of the forceps such that a mark on the balloon was aligned over a mark on the forceps. This mark, located 0.63 the distance from the fulcrum of the forceps to the tip, provided a consistent location for the user to rest their thumb and to apply pressure when crushing. This distance, between the fulcrum and the point of force application, was taken into account during the calculation of the crush force. In order to reflect only the force applied at the tip of the forceps, the recording software was adjusted to report a factor of 0.63 of the total detected applied force.

The force transducer was calibrated with standard weights before each use. A calibration device was constructed for this purpose. The instrumented forceps were placed in the center of the device such that a suspended weight could apply pressure to the balloon transducer and forceps in a manner similar to that of the thumb of the user during crushing. Using both 50- and 20-gram weights, the recording software was calibrated to detect 0.49 and 0.20 Newtons, respectively.

The baseline force involved in touching, but not crushing, the nerve with the forceps was recorded for 5 sec prior to crushing the nerve. While observing the nerve under direct light microscopy at 20× magnification, the primary investigator commenced crushing of the nerve by gradually increasing the force applied to the forceps at a rate of approximately 0.03 N/sec (Figures 4B,C). As the primary investigator was crushing, a second investigator monitored the CMAP response in real-time on-screen using the recording software. At a target decrease in CMAP amplitude of 70%–90% compared to baseline, the second investigator called out STOP. At this point, the primary investigator immediately ceased crushing by releasing the forceps. The total crush time was then recorded, in addition to the maximally applied force, and the rate of force increased over time.

### Statistical Analysis

CMAP amplitude data representing the duration before, during, and after each nerve crush trial was extracted from the LabChart recording software (ADInstruments LabChart for macOS, Version 8.1.17. Dunedin, New Zealand: ADInstruments Inc). Average CMAP amplitude values at each point of interest were determined as the average of five consecutive peaks. Maximal crush force was measured as the difference between the magnitude of touching the nerve and the maximal magnitude applied. Duration of crush was determined from the terminal time-point involved in touching the nerve to the moment of release of the forceps. The rate of graded increase in force application was determined by the slope of ΔNetwons/Δtime during the crushing interval. Data analysis was performed using SPSS (IBM SPSS Statistics for macOS, Version 24.0. Armonk, NY, United States: IBM Corp.) and Excel (Microsoft Excel for macOS, Version 16.47. Redmond, WA, United States: Microsoft Corporation).

### Histology

After completion of the CMAP testing and while the rat was still anesthetized, Karnovksy’s fixative (2.5% glutaraldehyde and 2.5% paraformaldehyde in 0.1 M phosphate buffer at 4°C) was applied to the tibial nerve at the lesion location. After 10 min, a 10 mm segment of the tibial nerve was removed and placed in fresh fixative in the refrigerator for at least 1 week. Selected nerve specimens were rinsed in saline, post-fixed in 1% OsO_4_ (with 1% potassium ferrocyanide) for 60–90 min, dehydrated in serial alcohols, cleared in propylene oxide, and embedded in Epon. Semi-thin sections (1 micron) of the tibial nerve cut in either the longitudinal or transverse plane were stained with methylene blue for light microscopy. The control lesion specimen was a comparable segment from either the contralateral unoperated side or a location proximal to the lesion. Slides containing selected stained tibial nerve sections at the injury site were uploaded to AperioImageScope (Version 12.4; Leica Biosystems; Wetzlar, Germany) software. A qualitative assessment of the crush lesion was to confirm the extent of the crush lesion with respect to the appearance of any intact axons and the longitudinal extent of the crushed zone.

Selected tibial nerve specimens were stained en bloc with 1% uranyl acetate in 50% ethanol for 1 h followed by further ultrathin sectioning and staining with Reynold’s lead citrate for 2 min for transmission electron microscopy (TEM). TEM was performed on a JEOL (JEM 1220) electron microscope (Tokyo, Japan). Qualitative TEM was used to correlate, confirm, and clarify the findings seen on light microscopy.

## Results

Of the 22 crush lesion trials performed, 19 were included in our analysis. Three trials were excluded due to technical failures or animal death before the contralateral side could be operated on. The average weight of the rats used was 268.9 ± 37.5 g.

**Figure 5:**
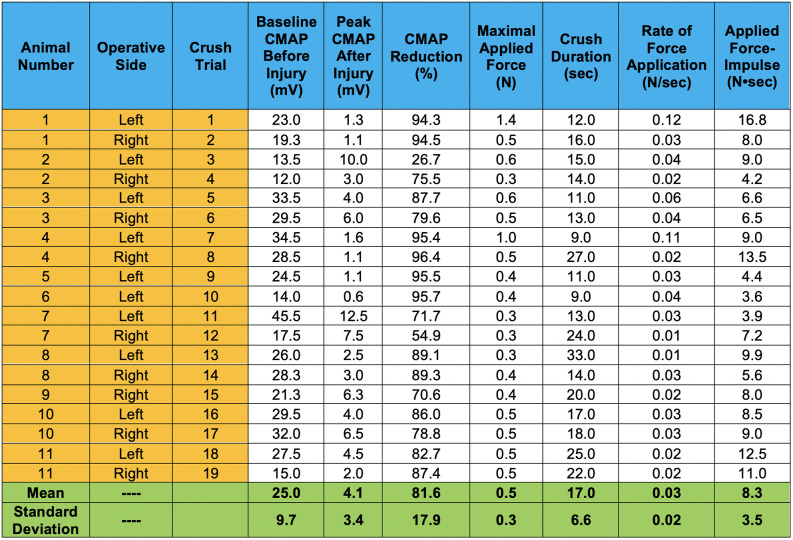
CMAP amplitude responses induced by our controlled compressive nerve lesions are shown. For each trial, CMAPs displayed represent values averaged from dual channel recordings, representing motor unit activity in both the lateral and medial gastrocnemius muscles. Peak CMAP values after injury represent the peaks immediately after the crushing forceps were released and extricated from the nerve. Crush duration represents only the time in which the graded force was applied. Rate of force application represents the slope of the graded force applied over the crushing interval.

The average baseline CMAP amplitude before nerve injury was 25 ± 9.7 mV. This decreased to 4.1 ± 3.4 mV immediately after the controlled crush injury was induced (measured at the moment the crushing forceps were released). This corresponds to an average decrease in CMAP amplitude of 81.6 ± 17.9%, as shown in Figure 5.

**Figure 6:**
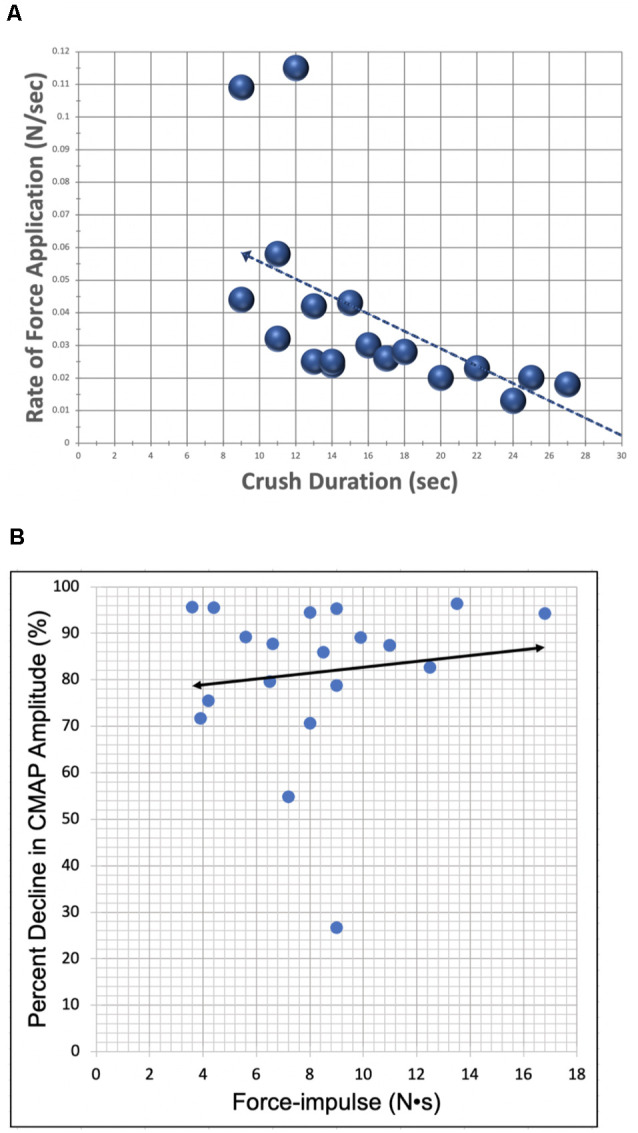
Scatter plots demonstrating correlation between lesion parameters. **(A)** The relationship between the rate of force application (y-axis) and crush duration (x-axis) for all crush trials (*N* = 19). Correlation (*r* = −0.609) was found to be statistically significant with a *p*-value = 0.006. **(B)** The relationship between percent decrease in CMAP amplitude (y-axis) and applied F-i (x-axis) for all crush trials (*N* = 19). Bivariate correlation between variables was found to be statistically insignificant, likely due to the relatively low power of this study.

The average duration of crush was 17 ± 6.6 sec. The average maximal applied force for all trials was 0.5 ± 0.3 N. This force was achieved by gradually increasing applied pressure at an average rate of 0.03 ± 0.02 N/sec over the duration of the crushing interval. The Pearson correlation coefficient between the rate of force application and crush duration is −0.61 at a significance level <0.01, indicating significance (Figure 6A). Thus, as the rate of applied force increased, overall crush duration was reduced. The correlation between applied F-i and the resulting percent decrease in CMAP amplitude was assessed for all trials (Figure 6B). The relationship between these crush parameters was found to be statistically insignificant: correlation of *r* = 0.127 (*p* = 0.604).

After crushing, CMAP was continually monitored for 5–20 min in 13 of 19 trials (68%), and for 5 min in 6 of 19 trials (32%). Following the initial injury-induced decrease in CMAP, eight of 19 trials (42%) demonstrated a partial and transient recovery of CMAP amplitude. The average maximal recovery in CMAP amplitude for these eight trials was 13.5 ± 10 mV, corresponding to an average recovery in CMAP amplitude of 45.7 ± 30.7%. This recovery was followed by a further decline, occurring within 2–4 min, for all eight trials. CMAP amplitude became zero in 13 of 19 trials (68%) by the end of the recordings. The average baseline CMAP amplitude at the end of the recordings was 2.4 ± 3.9 mV for all six trials which did not reach zero.

**Figure 7:**
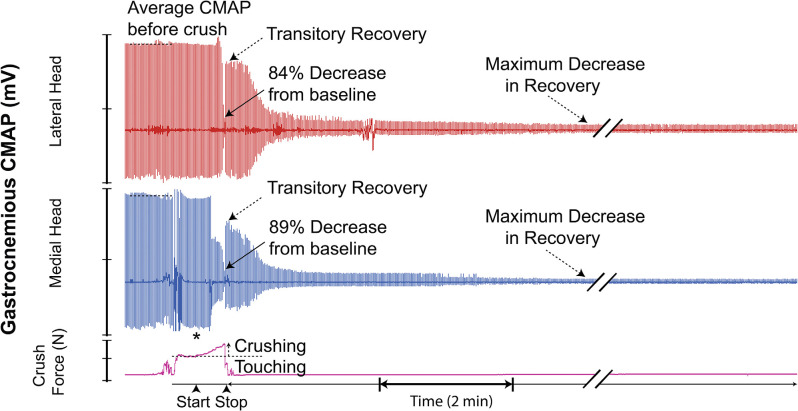
Recording demonstrating the typical two-channel CMAP response observed before, during, and after inducing a controlled compressive lesion on the tibial nerve. The force involved in touching the nerve and the subsequent graded increase in applied pressure required to induce axonotmesis is shown. This recording demonstrates transient partial CMAP amplitude recovery after sustaining the injury. The recovery was followed by a further decline. * = beginning of force increase.

**Figure 8:**
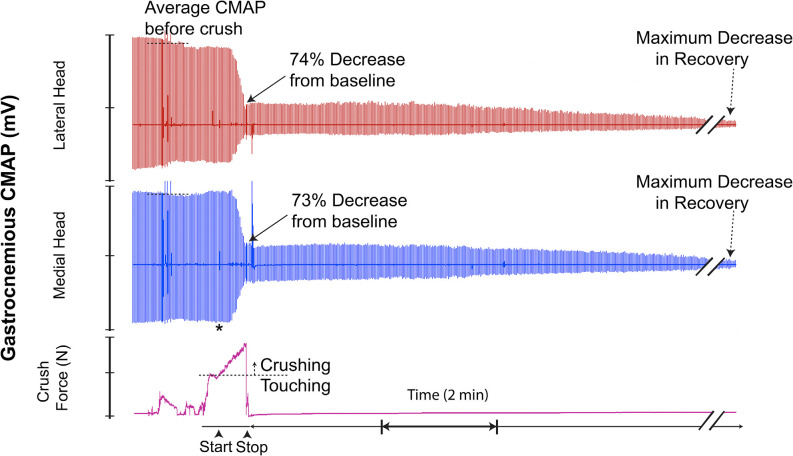
This recording demonstrates crush-induced decline in CMAP amplitude with no recovery and residual electrical activity (non-zero baseline) post-crush. * = beginning of force increase.

**Figure 9:**
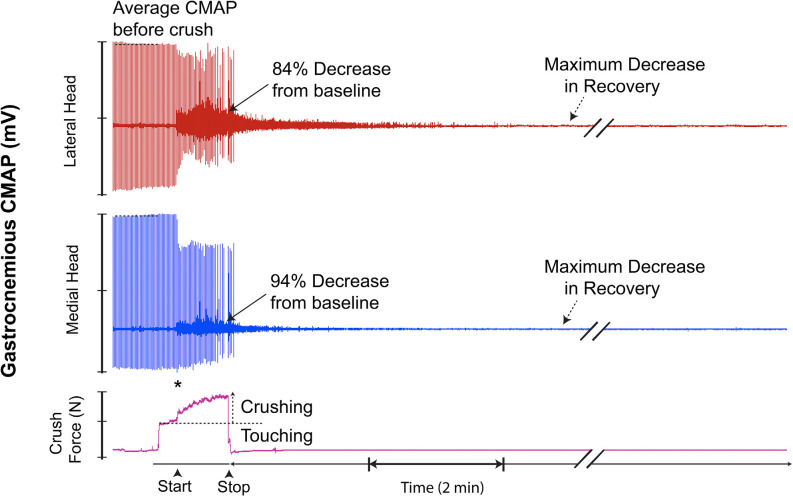
This recording demonstrates superimposed background EMG response. No transient recovery occurred after sustaining the injury. * = beginning of force increase.

**Figure 10:**
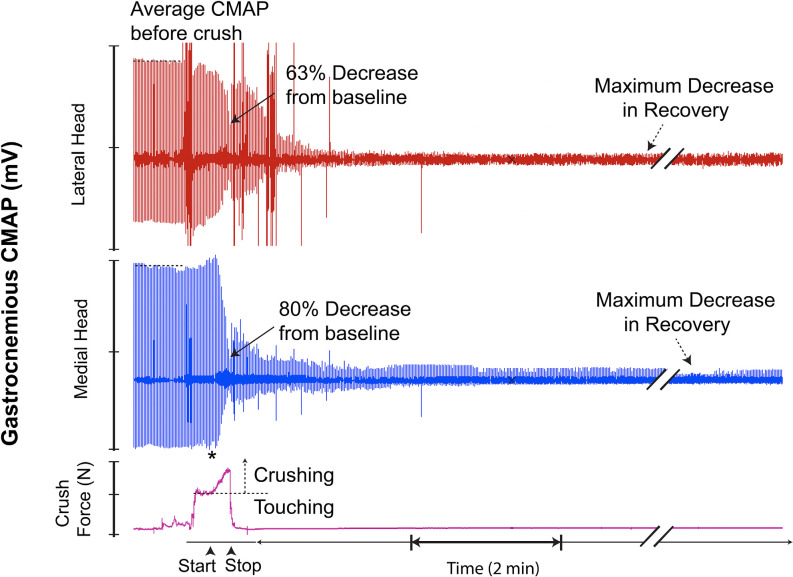
This recording demonstrates characteristics similar to the recording in Figure 7; however, with the addition of a transient recovery of CMAP immediately after crush termination, followed by further decline. * = beginning of force increase.

Twelve of 19 trials (63%) demonstrated a superimposed electromyographic background response during and after the crush event, with disappearance occurring within 4–8 min. Four two-channel CMAP recordings, which are representative of the typical CMAPs observed, are shown in Figures 7–10.

**Figure 11:**
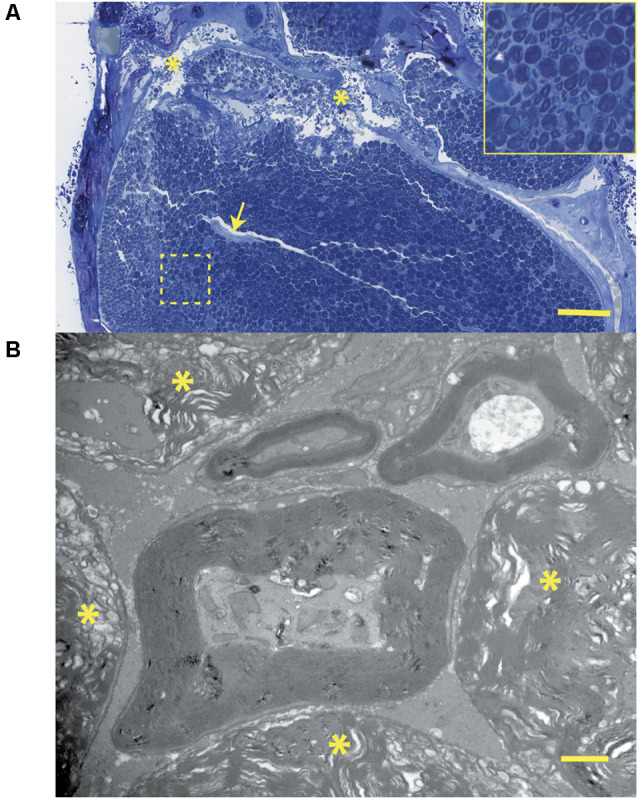
**(A)** Histology corresponding to the CMAP tracing of Figure 7: this transverse section through the crush zone shows the majority of myelinated axons are damaged. There are breaks in the perineurium (asterisks), a collapsed artery (arrow), and some superficial regions (near the periphery) containing small fibers that appear spared. The insert shows several axonal profiles that are non-myelinated. The two fascicles at the top supply the gastrocnemius muscle. Bar = 100 μm. **(B)** At the ultrastructural level, from the same tissue specimen shown in Figure 6A; the details of nerve fiber breakdown, progressing from small to large, are seen. Note that small fibers may appear “normal” and that axoplasmic changes precede those involving the myelin sheath. The non-myelinated axons (top) also appear normal. The many myelin figures (^*^) at the periphery all appear most advanced. Bar = 1 μm, insert is an enlargement of the dotted line region.

**Figure 12:**
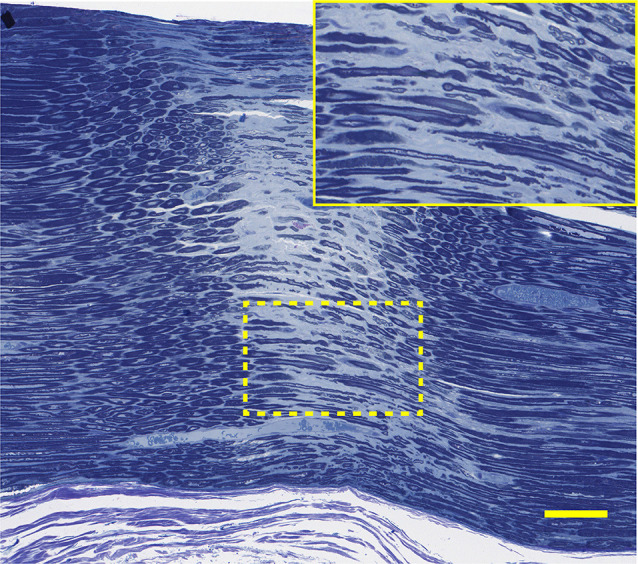
Histology corresponding to the CMAP tracing of 5D: this longitudinal section through the narrow nerve crush zone with distinct borders shows sparing of some small nerve fibers in the gap. Lesion width is ~300 μm. Bar = 100 μm, insert is an enlargement of the dotted line region.

**Figure 13:**
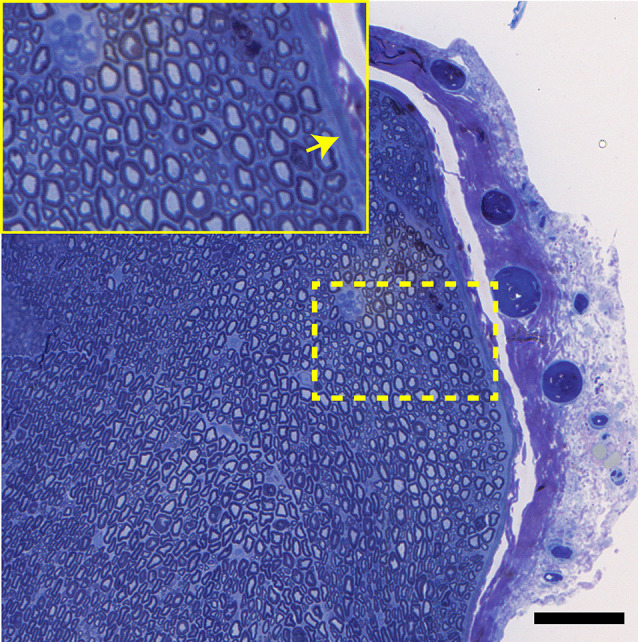
A transverse view of the normal tibial nerve. Note the normal appearance of the perineurium (arrow), as well as the large and small myelinated axons. Bar = 100 μm, insert is an enlargement of the dotted line region.

Light photomicrographs examining the crush zones of the tibial nerves in transverse and longitudinal sections are shown in Figures 11–13. A qualitative evaluation demonstrates that the majority of myelinated axons were damaged by the crush. However, small-sized fibers located near the surface (periphery) were preferentially spared, compared to larger more centrally located fibers. The perineurium and blood vessels were also affected in some cases (Figure 11A). Follow-up TEM confirmed the preferential sparing among small fibers and further demonstrated that the axoplasmic changes may occur prior to myelin sheath breakdown (Figure 11B). In addition, the small non-myelinated axons were also spared. It is unclear why the myelin is less sensitive to the crush forces compared to the axoplasm. Spared fibers were also seen crossing the lesion zone in the longitudinal sections (Figure 12). Measurement on these sections confirmed that the respective lesion width is ~250 μm, as suggested at the time of surgery (Figure 4B). Since the lesion is noticeably “crunched”, or contracted in on itself, the actual lesion width is likely larger than the measured distance.

When compared to their respective CMAPs, we notice that axonal sparing on histology is likely associated with the non-zero CMAP amplitude baseline seen several minutes post-crush. Hence, an incomplete crush of the tibial nerve may have been captured on the CMAP recordings as continued electrical signaling (non-zero baseline), post-crush.

## Discussion

In the present study, we demonstrate a technique by which real-time CMAP amplitude changes are used as an endpoint for conducting crush injuries to the rat tibial nerve. We also demonstrate how CMAPs can be used to characterize and quantify the loss of neuromuscular function sustained during and after controlled compressive injury. By instrumenting our crushing device with a force transducer, we were able to quantify the F-i of the induced lesion using data acquisition software. This allowed us to quantify all the parameters of the crush injury.

CMAPs are widely used for evaluating functional restitution after nerve injury in experimental animal models (Smith et al., 2000; Mikesh et al., 2018). In the study of axonotmesis, CMAPs are particularly useful for determining the course and extent of nerve regeneration and muscle reinnervation after injury (Navarro and Udina, 2009). To our knowledge, this is the first study to measure CMAP in real-time during actual induction of nerve injury, and to attempt use of the data gleaned as an objective endpoint, serving as a surrogate for crush effectiveness. By using CMAPs in this way, we have demonstrated a crushing technique that is controlled by a feedback loop, with the ultimate decision of whether to cease or continue applying pressure to the nerve being controlled by the human operator. The operator makes this decision based on the percent decrease in CMAP amplitude, which is displayed on screen in real-time and continually updated. As a proof of concept, we arbitrarily decided to continue crushing until observing a 70%–90% decline in CMAP amplitude from baseline. We continued recording CMAP amplitude changes even after the crushing event terminated in order to characterize and quantify the archetypal electrophysiological response of the tibial nerve immediately after sustaining crushing trauma. To our knowledge, this has not been described previously.

Appraisal of the crush parameters we used to induce nerve injury demonstrated that, for different crush trials, the F-i required to produce a ~70%–90% decrease in CMAP amplitude varied significantly. Although most nerves required ~8–10 N·sec to reach this endpoint, some nerves required as little as ~4 N·sec or as much as ~17 N·sec. Thus, the F-i required to produce a given electromyographic response is not fixed and seems to vary widely, even within the same animal species and when applied to the same nerve, as was the case in this study. Given that CMAP is itself a quantitative characterization of neuromuscular function, we can extrapolate that this variable response threshold to sustained trauma may also translate to corporeal variability, as observable behavioral motor deficits. In the context of producing axonotmesis, our results highlight the importance of not being too minimalist with regard to applied crush force and duration. Applying too small a force-impulse when conducting the nerve lesion may produce lesions with variable extents of injury.

Assessment of the electrophysiologic response patterns which occurred immediately after cessation of crushing revealed that, even when subjected to injuries of similar parameters, nerve CMAPs varied significantly in the temporal arrangement, propagation, and magnitude of electrical potential. While some crush trials displayed a transient recovery of CMAP amplitude immediately after cessation of injury conduction, others did not. Of those which displayed a transient recovery, some did so with relatively high electrical potentials, while others yielded only a few millivolts. Quantitatively, 42% of all analyzed crush trials demonstrated this temporary “rebound” in CMAP amplitude. The magnitude of recovery in CMAP amplitude ranged from 15% to 75% relative to each trial’s respective baseline. Interestingly, this recovery of electrical potential disappeared in all cases within 4 min after crush, and was then often followed by a further decline. A few minutes post-crush, some trials resulted in the complete abolishment of CMAP signal (at or near zero millivolts) while others retained residual signal propagation and reestablished a baseline at lower amplitudes. We believe that these residual electrical potentials may be related to extent of sparing within the lesion. Spared motor nerve fibers would theoretically continue propagating electrical potentials to their respectively innervated motor units. Given that CMAPs inherently reflect summations of all the evoked potentials produced by the motor units in the captured region (i.e., gastrocnemius), those axons which are disrupted by the crush would cease their contribution of electric potential. As a consequence, recorded CMAP amplitude would diminish in magnitude. However, it would not reach 0 mV if some spared nerve fibers maintained neuromuscular innervation. Given the complexity of the physiology involved, this relationship between acute residual electromyographic signaling and the extent of axonal sparing after crush injury requires further study.

As our resulting CMAP data suggests, the relationship between crush parameters, severity, and uniformity of nerve lesions, and captured electromyographic signal is further complicated by another observed phenomenon. Upon initiation of crushing, 63% of our crush trials demonstrated an emergence of erratic electrical signaling, akin to the appearance of electromyographic “noise”. Given that our CMAP recordings were obtained *via* an open and relatively invasive surgery, we were able to exclude potential sources of electrical interference (i.e., technical malfunctions, improper placement of electrodes, and animal death) quite easily. Moreover, this phenomenon, which can further be described as a brief, temporally dynamic CMAP amplitude change, with high frequency widely fluctuating electrical potentials, consistently appeared at the moment of crush initiation. This “noise” also consistently dissipated during or briefly after cessation of crushing.

Among the histological preparations assessed in the present study, we were surprised to see the consistent extent of axon sparing, especially among the small myelinated fibers. Compared to our previous study, the sequential breakdown of the axoplasm prior to myelin sheath was very similar to the Wallerian degeneration observed 5 days post-crush in the rat tibial nerve (Kerns et al., 2020). In the present study, the sequence of the observed changes was a result of injury itself, rather than Wallerian degeneration. In regards to the sparing observed, even a small proportion of fibers involved could have significance for subsequent regeneration and recovery. It has been shown that spinal cord injury (contusion) can have ~10% sparing, which translates to significant motor recovery (Kloos et al., 2005). This concept was first attributed to Andrew Blight in 1986 and deserves some reservations (personal communication). It remains to be shown that such possibilities also apply to the peripheral nervous system. The precise mechanism by which smaller axons are spared also needs to be determined. We propose that endoneurial tissue along with the large nerve fibers may play a protective role in producing this phenomenon, by cushioning. This is consistent in part, with the patterns of susceptibility given by Lundborg (2004) (pp. 49 and 58); motor > sensory, superficial > central, large > small. This observed sparing may involve sensory and non-myelinated axons as well as motor axons.

The importance of producing compressive lesions with quantitatively consistent parameters is highlighted by previous studies. In the context of axonotmesis, it has been reported that the extent of nerve recovery is dependent on the magnitude and duration of force applied during injury (Chen et al., 1992, 1993; Sarikcioglu and Ozkan, 2003). Despite this, no perfect standardized crushing method has been established. Moreover, no particular crushing device has become the mainstay for conducting crush lesions (Alvites et al., 2018).

Historically, various tools and methods have been used to induce compressive injury to peripheral nerves, each having advantages and disadvantages. The most elemental crush technique involves the use of simple or hemostatic forceps without modifications or instrumentation. Using these devices, a crush is produced by applying maximal compressive pressure to the nerve for 30–60 sec, or longer. Although this is a commonly used technique, its use experimentally is limited as it does not allow precise quantification of applied force. The use of tourniquets, applied around the limbs of an animal for minutes or hours, has been used to produce non-invasive compressive nerve injuries. However, owing to the difficulty in controlling the precise location and pressure applied to the nerve, the use of tourniquets has been deemed quantitative but indirect (Chen et al., 1992). The use of various commercially-available clamps has also been proposed. While these devices allow control of crush duration, they are limited by their inability to control applied force in a continuous manner and by the fact that it is not possible to obtain a graded compression through their use (Beer et al., 2001). Other innovative devices designed to experimentally compress nerves, such as compression boxes/chambers have also been proposed. While these devices allow induction of quantitatively controlled lesions, they must be specifically designed to suit the size of the animal model used as well as the nerve location (Rydevik and Lundborg, 1977; Chen et al., 1993). Despite the wide range of crushing devices available, the Dumont No. 5 micro-forceps seems to be the instrument of choice for conducting axonotmesis (Alvites et al., 2018).

In an attempt to address the heterogeneity of crushing instruments, previous studies have used various techniques to measure and report the approximate pressure applied by the crushing device onto the nerve (Alvites et al., 2018). However, only a few have sensorized their crushing device to allow real-time *in situ* measurement of applied force. Liu et al. (2020) used a miniature foil strain gauge sensor to achieve this, while Wandling et al. (2021) used a force-sensitive resistor (FSR). The former is known to be highly sensitive, accurate, and reliable for measuring applied force (Tamura et al., 2021). On the other hand, FSRs, which are composed of a piezoresistive material whose resistance decreases as applied mechanical pressure increases, are known to be limited by their relatively low accuracy (Schofield et al., 2016). Independent testing of the Flexiforce sensor used by Wandling et al. found that for forces up to 110 N, the sensors had an accuracy within ±0.5 N (Sadun et al., 2016; Parmar et al., 2017). The results of this study demonstrate that the average applied force necessary to produce axonotmesis is ~0.5 N, therefore, this margin of error may not be acceptable for this application. The method we used to quantify applied force utilized a medical-grade disposable blood pressure (BP) transducer. In accordance with the standards of the American National Standards Institute (ANSI), disposable BP transducers designed for clinical use, such as the one used in this study, must be accurate within a range of ±3%. A study by Gardner in which several commercially-available BP transducers were tested found that even the worst device was twice as accurate as required by the ANSI (Gardner, 1996). Having taken these factors into consideration, while also preferring an easily accessible, standardized, off-the-shelf product, we have successfully demonstrated the use of BP transducers for measurement of applied force in the context of nerve crush injury.

This study, and our proposed crush model, has a few notable limitations. Firstly, the power of our study was limited, involving only 11 animals and 19 crush trials (*N* = 19). Although a correlation between applied F-i during crush and a corresponding decrease in CMAP amplitude was detected, it was found to be statistically insignificant. This may be related to the limited sample size of our data. Secondly, we conducted all crush lesions manually—by hand. This intrinsically introduces an element of human error which is difficult to quantify, but which can be observed in our results. For instance, in several trials, we unintentionally under-shot or over-shot our target 70%–90% decrease in CMAP amplitude while conducting the crush. This is most likely attributable to delays in conveying and acting upon the real-time CMAP changes. Human error was also evident in producing the graded increase in applied pressure on the nerve, as the rate of force increase often varied from trial to trial. Despite this, we have shown an overall statistical correlation between the rate of increase in applied force and crush duration for all trials (Figure 6A). Potential future research could perhaps address the issue of human error by applying actuators to the crushing process. For example, using readily-available programmable microcontrollers and servos, a small, portable, highly accurate device can be developed to automate the crushing process through a closed-loop feedback system. This automated system would not only remove the error associated with a human operator but also make factors such as instrument positioning, response-time, and rate of force application more controllable and precise.

Thirdly, although our study provides preliminary data identifying the minimal threshold parameters of a crush lesion required to induce complete axonotmesis, with respect to force, duration, and extent—the precise parameters were not determined. This is a limitation of our experimental design as a more extensive histological study (e.g., quantitative and ultrastructural) would be required to determine the definitive threshold parameters in the rat tibial nerve, as well as the relationship between these parameters. Nonetheless, the primary reason we included histology in the present study was to determine the extent of the nerve lesion and the preliminary correlation with CMAPs. Moreover, although we could have easily applied our crush model to determine these lesion-specific threshold parameters, we chose not to ascertain them due to certain fundamental impediments which diminish the clinical relevancy and application of the results. For instance, determination of these parameters using a rat model alone is in itself a fundamental limitation because the extent of protection afforded by the nerve epineurium varies according to species being investigated and is greater in humans than in rats (Kerns, 2008; Alvites et al., 2018; Kerns et al., 2019). Threshold parameters also vary based on the specific nerve being investigated and on its anatomical location, with crushing parameters likely being greater at or near joints (Alvites et al., 2018). For these reasons, we considered the determination and characterization of threshold parameters to be outside the scope of our study.

Lastly, this study was also limited by the crushing device used—a Dumont No. 5 micro-forceps. Although the contact-width at the absolute tip was 100 microns, we conducted lesions ~2 mm down from the tip, where the contact width was ~300 microns. This was done with the idea of making the nerve easier to hold in between the clasps of the forceps during crushing. However, this was subject to slippage and may have resulted in variation in lesion width between trials. This highlights a disadvantage of using standard surgical forceps—the contact-width is not uniform and widens as one moves down from the tip. Therefore, if an investigator was attempting to produce a narrow lesion (≤100), and could not risk allowing slippage which would change the resulting lesion’s width, the use of surgical forceps would not be recommended.

Although the variability demonstrated by our results indicates that the use of CMAP amplitude decline may not be a reliable and objective endpoint for producing axonotmesis, we have shown that monitoring and recording CMAP data while concurrently inducing injury allows for the study and characterization of acute nerve responses and subsequent recovery. As previously alluded to, the modest crush parameters used in our study were found to inconsistently produce complete and uniform axonotmesis. Taken together with the variable electrophysiological responses observed during and after relatively equivalent injuries, we suggest that investigators maximize crush force and duration when producing axonotmesis. This may reduce the potential for partial sparing, the degree of which would be difficult to replicate.

Since nerve crush injury is recognized to be a clinically relevant entity, it can potentially benefit from a variety of interventions aimed at accelerating recovery or preventing functional declination. Interventions worthy of exploration could be aimed at reversing associated edema, regulating endoneurial pressure and blood flow, and especially, protecting nerves from the reactive oxygen species associated with reperfusion injury (Alvites et al., 2018). Experimentally conducting nerve injuries with concurrent monitoring of CMAP activity would allow characterization of the effects of a wide range of neurotrophic factors, growth factors, antioxidants, alkaloids, and pharmacological agents. For instance, real-time assessment of acute electrophysiological response post-injury would be particularly well-suited for exploration of the dramatic and early effects of fusogens, such as polyethylene glycol (PEG; Riley et al., 2015; Ghergherehchi et al., 2019). The study of the effects of PEG on nerve regeneration is of particular interest to researchers and has received significant coverage and discussion in recent literature (Riley et al., 2015; Bittner et al., 2016b; Ghergherehchi et al., 2019). Given PEG’s time-sensitive efficacy, elucidation of its acute effects on the restitution of nerve electrical conductivity and neuromuscular response may have considerable clinical implications (Bittner et al., 2016a). Moreover, with relatively few adaptations, CMAP data could be obtained *via* minimally-invasive (Nijhuis et al., 2011) or non-invasive techniques (Kerns et al., 1987; Wang et al., 2015) thus allowing collection of interval data, hours, days, or even weeks after the injury, without the risks associated with surgery. This paradigm can be applied to the study of all forms of nerve injury, including less severe lesions such as neuropraxia, more severe lesions such as neurotmesis, and to both complete or partial axonotmesis.

## Conclusion

Due to differences in tools and methods used, it’s often difficult to compare results obtained from different experimental investigations studying nerve axonotmesis. These differences are a manifestation of variations in crush parameters, including force exerted, crush duration, and contact-width. We have demonstrated a technique which overcomes some of these problems by using specially instrumented micro-forceps. Compared to other tools, the use of our crushing device allows quantification of all the parameters of the lesion. Moreover, we have shown that recording of CMAP during and after conduction of nerve lesions allows characterization of the acute nerve electrophysiological responses to trauma. Nerves have been shown to respond to acute trauma in a variety of ways, but with consistent patterns. Although too variable to be used as an objective endpoint for crushing, real-time *in situ* CMAP recordings may still offer some insight into the partiality of damage sustained and the sparing of axonal fibers post-traumatic injury. This model can also be used to test interventions aimed at enhancing subsequent regeneration and behavioral recovery.

## Data Availability Statement

The original contributions presented in the study are included in the article, further inquiries can be directed to the corresponding author.

## Ethics Statement

The animal study was reviewed and approved by the University of Illinois at Chicago Institutional Animal Care and Use Committee.

## Author Contributions

The authors confirm contribution to the article as follows: JK, MG, FA, and RD: study conception and design. MH, NB, DE, MG, FA, and JK: data collection. MH, NB, DE, and JK: analysis and interpretation of results. MH, NB, DE, RD, FA, MG, and JK: draft manuscript preparation. All authors reviewed the results and approved the final version of the manuscript. All authors contributed to the article and approved the submitted version.

## Conflict of Interest

The authors declare that the research was conducted in the absence of any commercial or financial relationships that could be construed as a potential conflict of interest.

## Publisher’s Note

All claims expressed in this article are solely those of the authors and do not necessarily represent those of their affiliated organizations, or those of the publisher, the editors and the reviewers. Any product that may be evaluated in this article, or claim that may be made by its manufacturer, is not guaranteed or endorsed by the publisher.
